# Supplementary material for: Febrile Seizures and Respiratory Viruses Determined by Multiplex Polymerase Chain Reaction Test and Clinical Diagnosis
Source: Children (Basel). 2020 Nov 17;7(11):234. doi: 10.3390/children7110234 (PMC7698419; doi:10.3390/children7110234)
Supplement: Supplementary file 1 [file children-07-00234-s001.pdf]

Supplementary Table S1. Comparison of seizure characteristics between children with positive and negative results for each respiratory virus

Factor	Bocavirus		Human herpes virus-6/7		Parainfluenza virus		Respiratory syncytial virus		Human metapneumovirus		Coronavirus	
	Negative (*n* = 119)	Positive (*n* = 19)	Negative (*n* = 219)	Positive (*n* = 16)	Negative (*n* = 123)	Positive (*n* = 15)	Negative (*n* = 126)	Positive (*n* = 12)	Negative (*n* = 131)	Positive (*n* = 7)	Negative (*n* = 132)	Positive (*n* = 6)
**Age, months, median (IQR)**	24 (14-33)	18 (15-24)	24 (17-35)*	14 (10-17)*	23 (15-33)	18 (15-25)	22 (14-31)	25 (21-28)	22 (15-31)	17 (10-38)	22 (15-31)	28 (16-35)
**Male sex**	73 (61.3)	13 (68.4)	139 (63.5)	10 (62.5)	77 (62.6)	9 (60.0)	79 (62.7)	7 (58.3)	80 (61.1)	6 (85.7)	82 (62.1)	4 (66.7)
**Complex febrile seizure**	48 (40.3)	7 (36.8)	85 (38.8)	3 (18.8)	49 (39.8)	6 (40.0)	52 (41.3)	3 (25.0)	52 (39.7)	3 (42.9)	52 (39.4)	3 (50.0)
**Type of seizure**												
**Generalized tonic-clonic**	99 (83.2)	18 (94.7)	183 (83.6)	11 (68.8)	105 (85.4)	12 (80.0)	107 (84.9)	10 (83.3)	110 (84.0)	7 (100.0)	112 (84.8)	5 (83.3)
**Generalized tonic**	14 (11.8)	0 (0.0)	23 (10.5)	3 (18.9)	12 (9.8)	2 (13.3)	13 (10.3)	1 (8.3)	14 (10.7)	0 (0.0)	13 (9.8)	1 (16.7)
**Others**	6 (5.0)	1 (5.3)	13 (5.9)	2 (12.5)	6 (4.9)	1 (6.7)	6 (4.8)	1 (8.3)	7 (5.3)	0 (0.0)	7 (5.3)	0 (0.0)
**Duration of seizure**												
**≤5 minutes**	98 (82.4)*	11 (57.9)*	177 (80.8)	14 (87.5)	96 (78.0)	13 (86.7)	97 (77.0)	12 (100.0)	104 (79.4)	5 (71.4)	105 (79.5)	4 (66.7)
**≤15 minutes**	10 (8.4)	8 (42.1)	26 (11.9)	2 (12.5)	16 (13.0)	2 (13.3)	18 (14.3)	0 (0.0)	16 (12.2)	2 (28.6)	17 (12.9)	1 (16.7)
**>15 minutes**	11 (9.2)	0 (0.0)	16 (7.3)	0 (0.0)	11 (8.9)	0 (0.0)	11 (8.7)	0 (0.0)	11 (8.4)	0 (0.0)	10 (7.6)	1 (16.7)
**Number of episodes of seizure within 24 hours**												
**1**	79 (66.4)	14 (73.7)	151 (68.9)	13 (81.3)	83 (67.5)	10 (66.7)	84 (66.7)	9 (75.0)	89 (67.9)	4 (57.1)	89 (67.4)	4 (66.7)
**2**	32 (26.9)	5 (26.3)	57 (26.0)	3 (18.8)	33 (26.8)	4 (26.7)	36 (28.6)	1 (8.3)	35 (26.7)	2 (28.6)	35 (26.5)	2 (33.3)
**≥3**	8 (6.7)	0 (0.0)	11 (5.0)	0 (0.0)	7 (5.7)	1 (6.7)	6 (4.8)	2 (16.7)	7 (5.3)	1 (14.3)	8 (6.1)	0 (0.0)
**Number of seizure attacks**												
**1**	51 (42.9)	12 (63.2)	93 (42.5)*	13 (81.3)*	57 (46.3)	6 (40.0)	57 (45.2)	6 (50.0)	59 (45.0)	4 (57.1)	60 (45.5)	3 (50.0)
**≥2**	68 (57.1)	7 (36.8)	126 (57.5)	3 (18.8)	66 (53.7)	9 (60.0)	69 (54.8)	6 (50.0)	72 (55.0)	3 (42.9)	72 (54.5)	3 (50.0)
**Time interval between seizure occurrence and fever onset** [a]												
**Concurrent**	15 (12.8)	1 (5.6)	23 (10.6)	1 (6.3)	14 (11.6)	2 (14.3)	16 (12.9)	0 (0.0)	16 (12.5)	0 (0.0)	16 (12.4)	0 (0.0)
**<24 hours**	79 (67.5)	13 (72.2)	158 (73.1)	10 (62.5)	83 (68.6)	9 (64.3)	83 (66.9)	9 (81.8)	88 (68.8)	4 (57.1)	87 (67.4)	5 (83.3)
**<72 hours**	22 (18.8)	4 (22.2)	32 (14.8)	5 (31.3)	23 (19.0)	3 (21.4)	25 (20.2)	1 (9.1)	23 (18.0)	3 (42.9)	25 (19.4)	1 (16.7)
**≥72 hours**	1 (0.9)	0 (.0)	3 (1.4)	0 (0.0)	1 (0.8)	0 (0.0)	0 (0.0)	1 (.1)	1 (0.8)	0 (0.0)	1 (0.8)	0 (0.0)
**Abnormal EEG result**	3/48 (6.3)	0/9 (0.0)	7/89 (7.9)	0/8 (0.0)	3/51 (5.9)	0/6 (0.0)	3/54 (5.6)	0/3 (0.0)	3/54 (5.6)	0/3 (0.0)	3/56 (5.4)	0/1 (0.0)
**Abnormal brain MRI result**	1/28 (3.6)	0/2 (0.0)	2/54 (3.7)	0/3 (0.0)	1/27 (3.7)	0/3 (0.0)	1/27 (3.7)	0/3 (0.0)	1/27 (3.7)	0/3 (0.0)	1/30 (3.3)	0/0
**Family history of febrile seizures**	38 (31.9)	7 (36.8)	70 (32.0)	6 (37.5)	38 (30.9)	7 (46.7)	42 (33.3)	3 (25.0)	41 (31.3)	4 (57.1)	44 (33.3)	1 (16.7)
**Subsequent diagnosis of epilepsy**	5 (4.2)	1 (5.3)	9 (4.1)	0 (0.0)	5 (4.1)	1 (6.7)	5 (4.0)	1 (8.3)	6 (4.6)	0 (0.0)	5 (3.8)	1 (16.7)

IQR: interquartile range; EEG: electroencephalography; MRI: magnetic resonance image. [a] Determined in 135 episodes. * $p < 0.05$ (comparison between children with positive and negative results for a virus in each respiratory virus)
